# Effect of contrast‐enhanced CT scans on heterogeneity corrected dose computations in the lung

**DOI:** 10.1120/jacmp.v7i4.2240

**Published:** 2006-11-28

**Authors:** Nichola A. Burridge, Carl G. Rowbottom, Paul A. Burt

**Affiliations:** ^1^ North Western Medical Physics Christie Hospital NHS Trust Wilmslow Road Manchester M20 4BX United Kingdom; ^2^ Department of Clinical Oncology Christie Hospital NHS Trust Wilmslow Road Manchester M20 4BX United Kingdom

**Keywords:** radiotherapy, dose calculations, contrast‐enhanced CT

## Abstract

The aim of this study was to investigate and, if possible, compensate for the effect of intravenous contrast‐enhanced CT scans on the treatment planning dose distributions for lung patients. The contrast and noncontrast CT scans of 3 patients were registered, and the effect of contrast on the Hounsfield units (HU) was assessed. The effect of contrast was then simulated in the CT scans of 18 patients receiving radiotherapy of the lung by modification of the CT numbers for relevant sections of noncontrast‐enhanced CT scans. All treatment planning was performed on the Pinnacle^3^ planning system. The dose distributions computed from simulated contrast CT scans were compared to the original dose distributions by comparison of the monitor units (MUs) for each beam in the treatment plan required to deliver the prescribed dose to the isocenter as well as a comparison of the total MUs for each patient, a percentage change in required MUs being equivalent to a percentage change in the dose. A correction strategy to enable the use of contrast‐enhanced CT scans in treatment planning was developed, and the feasibility of applying the strategy was investigated by calculating dose distributions for both the original and simulated contrast CT scans. A mean increase in the overall patient MUs of 1.0 ± 0.8% was found, with a maximum increase of 3.3% when contrast was simulated on the original CT scans. The simulated contrast scans confirmed that the use of contrast‐enhanced CT scans for routine treatment planning would result in a systematic change in the dose delivered to the isocenter. The devised correction strategy had no clinically relevant effect on the dose distribution for the original CT scans. The application of the correction strategy to the simulated contrast CT scans led to a reduction of the mean difference in the overall MUs to 0.1 ± 0.2% compared to the original scan, demonstrating that the effect of contrast was eliminated with the correction strategy. This work has highlighted the problems associated with using contrast‐enhanced CT scans in heterogeneity corrected dose computation. Contrast visible in the CT scan is transient and should not be accounted for in the treatment plan. A correction strategy has been developed that minimizes the effect of intravenous contrast while having no clinical effect on noncontrast CT scans. The correction strategy allows the use of contrast without detriment to the treatment plan.

PACS number: 87.53.Tf

## I. INTRODUCTION

Three‐dimensional conformal radiotherapy has led to a more accurate delivery of radiotherapy and the possibility for dose escalation and lower toxicity. However, to fully gain from this, accurate delineation of the gross tumor volume (GTV) is essential. In the case of the lung, large margins are added to the GTV to compensate for breathing and cardiac motion, leading to increased toxicity as a larger volume of normal lung tissue is irradiated. The accurate definition of the GTV in this case is important in minimizing the overall treatment volume. However, studies have shown that this can be difficult, and GTV definition in the lung has been found to vary significantly among professions, centers, and levels of experience.^(^ ^1^ ^,^ ^2^ ^)^

Modern 3D treatment‐planning systems use the density information within the CT scan to account for the different tissue densities within the body. This is to ensure that the dose calculated will be a true representation of the dose distribution within the body. The attenuation of radiation at therapeutic energies is dominated by Compton scatter, which is dependent on electron density. The Hounsfield units (HU) from the CT scan are converted to either electron or physical densities via a CT number to density conversion table within the planning system. From this, the mass attenuation coefficient can be found using a look‐up table; the degree of attenuation can therefore be predicted. In the case of the Pinnacle treatment‐planning system, the fluence attenuation table is stored as a function of radiological depth, physical density, and off‐axis angle. The physical density allows the attenuation of the fluence to be based on the mass attenuation of the specific material within a given voxel. This correction is particularly important in lung cancer treatment planning due to the low density of lung tissue. If this is not corrected for in the dose calculation, the delivered dose can be as much as 15% greater than expected.^(3)^ Equally, if the CT dataset is an inaccurate representation of the tissue density due to artifacts resulting from markers, prosthetic hips, or contrast media, calculation errors may be introduced when a heterogeneity correction is applied.^(4)^

Lung cancer is the most common cause of cancer death in the developed world. Both small cell and non‐small‐cell lung cancers tend to involve central structures within the thorax by local invasion and lymph node metastases. As part of diagnosis and staging, CT scans of the thorax are performed using intravenous contrast to differentiate the numerous vascular structures from other tissue that may be involved with the cancer. When the patient attends at a later date for treatment planning, this advantage is lost if contrast material is not used as part of the radiotherapy planning CT scan. Contrast‐enhanced CT scans can be used to aid in the definition of the GTV to distinguish the tumor from vascular structures, particularly in the mediastinum.^(2)^ However, the use of contrast‐enhanced CT scans for treatment planning where heterogeneities are accounted for could adversely influence the dose distribution, the magnitude of which must be investigated prior to the safe implementation of this technique. It should be remembered that the contrast is transient in nature and will not be present at the time of treatment.

Contrast agents are made up of elements with high attenuation coefficients peaks in the diagnostic energy range, such as iodine. These peaks result in a high HU, which is interpreted as higher‐density tissue on the CT scan. The dose distribution computed by the planning system may therefore be misleading. A previous study has shown that the use of contrast media in CT scanning does lead to a change in the dose calculated by a 3D treatment‐planning system. If the absolute volume containing contrast agent and the corresponding increase in HU are limited, the difference will be 1% to 3%.^(5)^

A possible method to avoid this calculation error is to register pre‐ and postcontrast scans. This has the advantage of increased visualization, and the treatment could be planned on the noncontrast scan to ensure an accurate dose calculation. This can be done with accuracy for other sites that are anatomically stable, such as brain tumors. However, the movement associated with the lungs due to respiration and cardiac motion makes this less feasible. With fast CT scanning the breathing phase of each slice is slightly different than the previous and next slices. Consecutive scans of the patient are therefore unlikely to be easily registered unless the same phase of the breathing cycle is present at the start of the acquisition for both scans and the patient breathing is consistent between them. Another possibility is to ignore heterogeneity in the dose calculation, but, as already stated, the effect on the dose distribution is unacceptable in the lungs.^(6)^ A third option is to use the contrast‐enhanced CT scan for planning, but to develop a correction strategy to account for the higher HU of the contrast agent. This is the option we chose to pursue.

The aims of this study were to investigate the magnitude of the effect of an intravenously administered contrast agent on the dose distributions computed by a 3D treatment‐planning system, to develop a strategy for using intravenous contrast scans without significantly altering the dose distribution, and, finally, to quantify the residual error as a result of using this strategy.

## II. METHOD

The treatment plans of 18 patients who had undergone radiotherapy for lung cancer at the Christie were collated for this investigation. The 18 patients were chosen as a representative sample of differing tumor size and location as expected from clinical experience. All had been planned on the Pinnacle^3^ (Philips Medical Systems, Madison, WI) treatment‐planning system and the dose distribution computed with a heterogeneity correction applied. Pinnacle^3^ uses a collapsed cone convolution superposition algorithm that corrects for both primary and secondary scattered radiation.^(7)^ This is a more accurate heterogeneity correction compared to the simple equivalent pathlength algorithm, which tends to overestimate the dose in low‐density mediums.^(8)^

**Figure 1:**
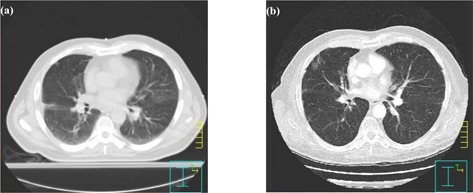
RTP and (b) contrast‐enhanced diagnostic CT scans for one of the patients in the study. These highlight some of the main differences between the scanning protocols, including the curved couch surface and an increase in the lung volume for the diagnostic scan. The presence of the contrast is also clear in the mediastinum on the diagnostic contrast‐enhanced CT scan.

The initial part of the investigation evaluated the effect of using a contrast‐enhanced CT scan on the dose calculation. For three patients in the investigation, diagnostic quality contrast‐enhanced CT scans had been acquired as part of the patients' management and were available in DICOM format. These were acquired on the GE Medical Systems Lightspeed Plus CT scanner at 120 kV/210 mA. The contrast was intravenously injected using an Envision CT™ injection system and the scan acquisition initiated after a predefined threshold CT number had been reached. This threshold was set at 50 HU above the baseline value in a region of interest within the ascending thoracic aorta. Two hundred milliliters of Omnipaque® contrast agent containing 140 mg/mL of iodine was used as standard contrast. The contrast‐enhanced diagnostic scans were exported to Pinnacle for comparison to the corresponding radiotherapy treatment‐planning (RTP) scans performed on either the GE Medical Systems Lightspeed or GE Medical Systems CTI scanner at 120 kV. The relationship between HU and physical density for the two scanners has been investigated and found to be equivalent, allowing a comparison of density to be made irrespective of the CT scanner used. An example of the difference between the two scans is shown in Fig. 1. Using the image fusion option within the Pinnacle software, the CT datasets were registered. This was not an accurate registration because the setup of the patient for the two scans was very different in terms of breathing protocol, arm position, and couch; however, it was adequate for the purpose of comparing MUs in similar regions of the two scans. The lungs, mediastinum, and a region of soft tissue in the anterior and posterior region of the body were outlined on the registered CT scans and compared in terms of HUs. Outlining was carefully performed to allow a robust comparison of density in similar patient regions for contrast and noncontrast CT scans. In particular, the outlines were not drawn too close to tissue interfaces and boundaries.

**Figure 2:**
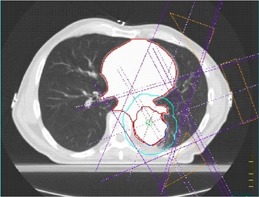
RTP CT scan with the mediastinum, GTV, and planning target volume outlined. The CT numbers in the mediastinum and GTV have been overridden, corresponding to an increase of 200 HU, representing a worst‐case scenario. In this particular example the left anterior oblique beam passes through the contrast‐enhanced mediastinum, causing increased attenuation of the beam.

To simulate the effect of using a contrast CT scan in the planning stage, the density of the mediastinum in the RTP scan was increased corresponding to the typical increase in HU seen on the contrast scans. The density of the GTV was also increased by the same amount. A similar degree of enhancement was assumed due to the increased vascularization of the tumor and a worst‐case scenario. The mediastinum was outlined in each of the 18 patients, and the mean HU was found from the Pinnacle software. The CT number in the mediastinum region and the GTV was then increased in turn by 50 HU, 100 HU, and 200 HU to include the range of HU increase seen in a contrast‐enhanced CT scan and an estimation of an expected upper limit for HU enhancement given the limited available data. This range is due to the variation in circulation and heart rates of the patients. This was achieved by overriding the CT number for the region of interest within Pinnacle with the new value. This is illustrated in Fig. 2. Here, the mediastinum and GTV are outlined and the CT number overridden corresponding to an increase of 200 HU in the CT number. The dose distribution was calculated with the full heterogeneity correction applied. The MUs to prescribe the required dose to the isocenter (ranging from 4500 cGy to 5500 cGy in 20 fractions) for each density were then compared and ultimately prescribed to the original CT dataset to evaluate the effect on the dose distribution. No change was made to the original treatment plan in terms of beam angles, field shapes, or wedge angles at any stage during the investigation.

The dose distributions within the 80% isodose were compared using a software tool developed in‐house^(9)^ based on the gamma method of Low et al.^(10)^ as implemented by Depuydt et al.^(11)^ This method enables dose distributions to be examined simultaneously for dose difference and distance to agreement between isodose lines. Various gamma criteria (1%/mm to 5%/mm) were investigated for simulated contrast enhancements of 50 HU, 100 HU, and 200 HU, and the percentage of pixels failing within the 80% isodose was calculated. The 80% isodose surface was chosen because it is a stable isodose that shows minimal variation between dose distributions computed using different heterogeneity corrections. It also represents the high dose volume of interest in and around the planning target volume, where significant changes in the dose distribution may lead to implications in terms of tumor control.

**Figure 3:**
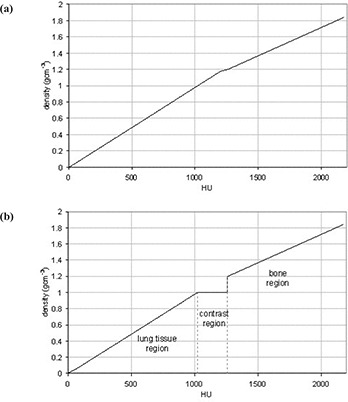
CT to density tables used in the investigation (a) clinical CT to density table and (b) modified CT to density table, where density values from $1\;g\cdot {\text{cm}}^{-3}$ to $1.2\;g\cdot {\text{cm}}^{-3}$ have been set to $1\;g\cdot {\text{cm}}^{-3}$

A strategy was developed in order to minimize the effect of using a contrast‐enhanced CT scan in the radiotherapy planning stage without significantly altering the dose distribution computed by the planning system. The CT to density conversion table used clinically was altered to reduce the effect of the increased density due to the contrast agent. Tissues with a density between $1\;g\cdot {\text{cm}}^{-3}$ and 1.2 g·cm^‐3^ were set equal to a density of $1\;g\cdot {\text{cm}}^{-3}$. This range covers the increased densities resulting from simulating contrast in the CT scan. At densities above $1.2\;g\cdot {\text{cm}}^{-3}$, the CT to density table corresponded to its original clinical values (bone region). This will correct the effect of the artificially high‐density tissue regions arising due to the contrast media, but would also allow a heterogeneity correction for the effects of high‐density materials such as bone. The two CT to density tables used in the study are shown in Fig. 3. The effect of using this correction strategy was investigated by comparing the dose distributions resulting from using the modified CT to density table on the noncontrast CT scan.

The final stage was to investigate the effect of using the correction strategy on the simulated contrast‐enhanced CT scans. The dose distribution for each patient (with the varying degrees of HU increase) was computed using the modified CT to density table. The MUs to prescribe the required dose were then compared to those for the original plan. Finally, the dose distribution analysis using the gamma evaluation software was repeated to ensure that any variations in the dose distribution resulting from the use of contrast‐enhanced scans were reduced by applying this correction strategy.

## III. RESULTS

### A. Effect of contrast agent on the CT scan

For the three representative patients the comparison of contrast with noncontrast CT scans showed that the difference in HU for the majority of soft tissue was negligible between the two scans. However, the HU for the mediastinum increased in the contrast scan by an average of approximately 100 HU, ranging from 60 HU to 130 HU. The HU of the lungs was found to decrease by an average of 70 HU in the contrast scans.

Because the soft tissue showed no significant increase in HU in the contrast‐enhanced scan, the soft tissue density of the RTP scan was not altered in the investigation. A slight decrease in HU was seen for the lung tissue, but this must be treated with caution. As already stated, the setups of the diagnostic scan and the RTP scan are very different. One of the main differences is in the breathing protocol; during the RTP scan, the patients are free to breathe, whereas the patients are asked to hold their breath at inhalation for the diagnostic scan. This results in a larger volume of air within the lungs for the diagnostic scan, hence decreasing the apparent density of the lung tissue on the scan. This is the likely reason for the apparent decrease in the lung density rather than any effect of the contrast administered. In the treatment setting the patient will be breathing freely; therefore, no alteration to the lung tissue density was made.

Increasing the CT number of the mediastinum and GTV in the range 50 HU to 200 HU was sufficient to cover the range of values found in the limited datasets available with contrast media and to investigate the effect of using higher concentrations of contrast media in the scans.

### B. Effect of using contrast‐enhanced CT scans in the planning stage

In the noncontrast scans, the densities of the mediastinum and the GTV were overridden to simulate the presence of contrast agent in the CT scan. For 3 of the 18 patients in the study, the clinician had not outlined the GTV. However, in all of these cases, the GTV was within the mediastinum. Because the densities of the mediastinum and the GTV were found to be similar (mean densities of $0.99\;g\cdot {\text{cm}}^{-3}$ and $0.94\;g\cdot {\text{cm}}^{-3}$, respectively), the effect of contrast on the GTV was still taken into account by simply increasing the density of the mediastinum in these cases.

**Figure 4:**
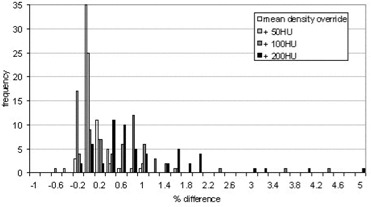
Histogram showing the percent difference (overridden/original) in the MUs units calculated for individual beams when the densities of the mediastinum and GTV are overridden to simulate contrast

**Figure 5:**
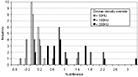
Histogram showing the percent difference (overridden/original) in the MUs calculated for each patient when the densities of the mediastinum and GTV are overridden to simulate contrast

The CT numbers of the mediastinum and the GTV were overridden with their mean CT number, and then the density of each was increased corresponding to an increase of 50 HU, 100 HU, and 200 HU. The calculated MUs were then compared to those from the original treatment plan with no contrast. Histograms showing the percentage difference between the MUs calculated using the various density overrides and the original plan are shown for each beam in Fig. 4 and the total for each patient in Fig. 5. The results are also summarized in Tables 1and 2.

**Table 1:** Individual beam results for the difference in MUs due to increasing the densities of the mediastinum and GTV

		Density override		
% difference	mean	+ 50 HU	+ 100 HU	+ 200 HU
average	0.1	0.1	0.7	1.0
SD	0.2	0.4	0.8	1.0
maximum	1.0	1.6	4.5	4.9

**Table 2:** Overall patient results for the difference in MUs due to increasing the densities of the mediastinum and GTV

		Density override		
% difference	mean	+ 50 HU	+ 100 HU	+ 200 HU
average	0.1	0.1	0.7	1.0
SD	0.1	0.4	0.6	0.8
maximum	0.3	1.3	2.2	3.3

In general, the percentage difference in MUs prescribed increased as the density was increased, as expected. This is due to the increased attenuation of the beam as it passes through higher‐density tissue. More MUs are therefore required to achieve the prescribed dose to the isocenter. To summarize the results for individual beam data, little difference was seen with an increase of 50 HU (average percentage difference 0.1±0.4%), but an increase of 100 HU had a larger effect on the MUs with an average percentage difference of 0.7 ± 0.8% and a maximum difference of 3.5%. An increase of 200 HU led to an average 1.0 ± 1.0% increase in the number of MUs prescribed with a maximum increase of approximately 5%. From the range of increased HU values of 60 to 130 seen in the diagnostic scans, it would appear that the contrast‐enhanced CT scans can be used for treatment planning without significant changes to the dose distribution or the MUs prescribed.

The diagnostic scans used in this study contained small amounts of contrast to better visualize the vasculature of the patient. If higher concentrations of contrast were used in practice to aid visualization, then the increase in the HU would be higher. This highlights the potential need to develop a correction strategy to account for the effect of contrast if it is to be used in the planning stage, depending on the concentration of contrast used during scanning. The maximum difference for each patient is less than for individual beams because it is unlikely that all the beams associated with a patient will be affected to a maximum extent (i.e., all passing through the mediastinum), so the effect of contrast is diluted. Despite this, the average increase in MUs is still approximately 1% with a maximum increase of over 3% when a 200 HU enhancement of the mediastinum and GTV is observed.

**Figure 6:**
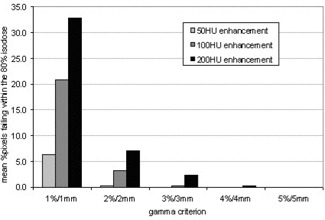
Histogram showing the mean percentage of pixels within the 80% isodose that failed the gamma criterion using gamma criteria of 1, 2, 3, 4, and 5%/mm

The difference in the MUs prescribed to deliver a dose to the isocenter with contrast present in the CT scans provides a measure of the potential overdose to the prescription point at treatment because the density predicted by the planning system will not be present at that time. However, the difference in the MUs does not offer an indication of the variation in the dose distribution due to the presence of contrast. In order to examine this variation, gamma evaluation was used to determine differences in the dose distributions between the original plan and the plan created when the MUs from the plan with the contrast were applied to the original noncontrast scan. The results are summarized in Fig. 6. As expected, the percentage of pixels within the 80% isodose failing the gamma criterion increased as the simulated enhancement increased and the gamma criterion increased.

### C. Effect of modified CT to density table on standard plans

When the mediastinum and GTV were outlined, the ranges of density for contrast and noncontrast scans were taken into account when designing a modified CT to density table. The density range was found to be $1.01\;g\cdot {\text{cm}}^{-3}$ to $1.18\;g\cdot {\text{cm}}^{-3}$, usually describing tissue from fat and water $\left(1.0\;g\cdot {\text{cm}}^{-3}\right)$ through to muscle $\left(1.1\;g\cdot {\text{cm}}^{-3}\right)$. Higher‐density bone such as the spine was found to have a mean density of around $1.3\;g\cdot {\text{cm}}^{-3}$.

**Figure 7:**
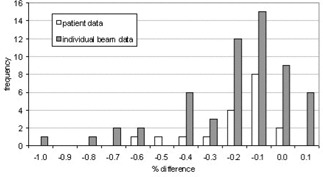
Histogram showing the distribution of percent differences between the MUs calculated using the two different CT to density tables in noncontrast‐enhanced scans

The mean difference between the MUs for noncontrast scans calculated using the clinical CT to density table and those calculated using the modified CT to density table devised for this project was −0.2±0.2% for individual beams and −0.2±0.2% for the overall patient MUs. The results are shown in Fig. 7. The MUs calculated for all beams were within 1% of their original value, and the overall maximum difference for an individual patient was −0.6%. This demonstrates that using the modified CT to density table on the noncontrast CT scans has negligible effect on the dose distribution when compared to the original plan. Therefore, the correction strategy can be further explored and tested on the simulated contrast CT scans.

### D. Effect of modified CT to density table on plans with simulated contrast‐enhanced scans

**Figure 8:**
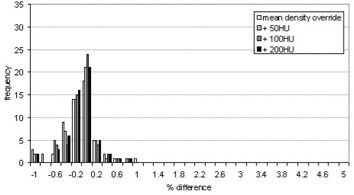
Histogram showing the percent difference in MUs resulting from using the modified CT to density table on the contrast scans for individual beams

**Figure 9:**
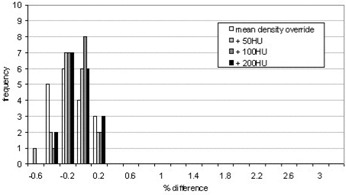
Histogram showing the percent difference in MUs for each patient resulting from using the modified CT table on the contrast scans compared to the original plan

The results of applying the modified CT to density table are presented on an individual beam basis as well as an overall difference for each patient. The results shown in Figs. 8 and 9 are presented on the same scale as the previous results in Figs. 4 and 5 to highlight the improvement made by using the modified CT table over the original clinical CT table. The average percentage difference was within −0.2% for both the individual beams and the overall patient with maximum values of −1.0% and −0.5%, respectively. There was no variation in the differences seen with increasing HU, demonstrating that the modified CT to density table achieves its goal in eliminating the effects of contrast.

**Figure 10:**
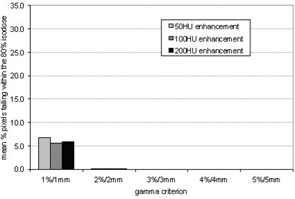
Histogram showing the mean percentage of pixels within the 80% isodose that failed the gamma criterion. The number of pixels failing has been significantly reduced by using the modified CT to density table.

The gamma evaluation also produced excellent results as shown in Fig. 10. The percentage of pixels that failed the gamma criteria was significantly reduced, with no pixels failing the 2%/mm criterion. Again, the results have been produced on the same scale as in Fig. 6 to fully demonstrate the effect of using the correction strategy. There is no dependence of dose distribution differences on the degree of enhancement simulated in the CT scan.

## IV. DISCUSSION

Ideally, contrast‐enhanced CT scans would be used to assist in delineating the GTV in carcinoma of the lung. However, due to the heterogeneity correction applied in the calculation of dose for this site, the presence of contrast introduces an error in the dose calculation that must be considered and corrected for if significant; otherwise, a systematic error will be introduced into the treatment at the planning stage. Using simulated contrast scans of the lung, we have shown that the error resulting from using a heterogeneity correction in the presence of contrast is as high as 5% for an individual beam. This assumes an increase of 200 HU in the CT number of the mediastinum and GTV, but even with only a 100 HU rise in the CT number a maximum increase of 4.5% was seen. However, if low concentrations of contrast media are used for the visualization, then the resulting dose distribution without any correction strategy will only contain approximately 1% error on average. Given the improvement in the delineation of the GTV that may be achieved with low concentrations of contrast, the introduction of a 1% systematic error in the dose distribution is likely to be clinically acceptable.

The MUs increased when the contrast CT scan was used because the CT numbers of the mediastinum and GTV were artificially high. This resulted in the dose algorithm assuming a greater attenuation of the incident radiation and calculating that more MUs would be required to deliver the prescribed dose to the isocenter.

The results highlight the error that could result from using a contrast scan in the dose calculation of the planning stage. This error may in fact be worse when a true contrast‐enhanced CT scan is used. In the simulated scans, only the mediastinum and GTV were overridden with an increased CT number. Due to outlining limitations and structure delineation, the CT number enhancement of all structures within the limited contrast scans available could not be assessed. Therefore, there may be areas of contrast uptake that have not been fully taken into account in this study. The range of HUs considered does, however, seem comparable to the findings of other studies.^(12)^

In order to use a contrast‐enhanced CT scan in the planning stage without significantly altering the dose computation, a correction strategy was developed that involved modification of the CT to density table in the planning system to eliminate the effect of the contrast agent on the dose calculation. The results were very promising when this modified CT table was applied to a noncontrast scan with the overall patient MUs within 0.6% of the MUs for the original plan for all 18 patients. The modified CT table also had the desired effect when it was applied to the simulated contrast scans. The differences between the MUs calculated and the MUs for the original plan were all within an acceptable clinical uncertainty of 1% for each individual beam as well as for the overall patient, reducing possible systematic errors of up to 5%. This means that a contrast‐enhanced CT scan may be used in the planning stage of the radiotherapy treatment without significantly affecting the delivered dose distribution with no contrast present.

This work has considered chest scans using contrast administered intravenously. A similar technique could therefore be used for intravenous contrast in other sites such as the brain. However, the presence of the blood brain barrier results in less contrast enhancement in the brain^(^ ^13^ ^,^ ^14^ ^)^; therefore, the dose computation would be less affected. Oral administration of contrast agent, for example, in enhancement of the bladder, produces a bolus distribution of enhancement with large HU increases. Often the density is similar to cortical bone, calcifications, or metal prostheses, so this cannot be ignored in the dose calculation without serious detriment to the resulting dose distribution. This is a separate problem, and a correction strategy such as described here would not be suitable in this case.

## V. CONCLUSIONS

This work has highlighted the problems associated with using contrast scans when a heterogeneity correction is applied in the dose calculation. A correction strategy has been developed that minimizes the effect of contrast on the dose computation. This has been shown to work well on simulated contrast scans and potentially allows the use of intravenous contrast CT scans for routine treatment planning in the thorax region. To take the investigation further, the next stage would be to assess the use of the correction strategy on true contrast scans of the lung. This will ensure that the strategy is suitable to be used clinically.

## References

[1] Giraud P , Elles S , Helfre S , et al. Conformal therapy for lung cancer: Different delineation of the gross tumour volume (GTV) by radiologists and radiation oncologists. Radiother Oncol. 2003; 62: 27–36. 10.1016/s0167-8140(01)00444-3 11830310

[2] Senan S , van Sörnsen de Koste J , Samson M , et al. Evaluation of a target contouring protocol for 3D conformal radiotherapy in non‐small cell lung cancer. Radiother Oncol. 1999; 53: 247–255. 10660205 10.1016/s0167-8140(99)00143-7

[3] Morrill SM , Langer ML , Lane RG , Rosen II . Tissue heterogeneity effects in treatment plan optimization. Int J Radiat Oncol Biol Phys. 1994; 30: 699–706. 7928503 10.1016/0360-3016(92)90958-k

[4] Williams G , Tobler M , Gaffney D , Moeller J , Leavitt D . Dose calculation errors due to inaccurate representation of heterogeneity correction obtained from computerized tomography. Med Dosim. 2002; 27: 275–278. 12521074 10.1016/s0958-3947(02)00147-4

[5] Ramm U , Damrau M , Mose S , Manegold KH , Rahl CG , Böttcher HD . Influence of CT contrast agents on dose calculations in a 3D treatment planning system. Phys Med Biol. 2001; 46: 2631–2635. 11686279 10.1088/0031-9155/46/10/308

[6] Morrill SM , Langer ML , Lane RG , Rosen II . Tissue heterogeneity effects in treatment plan optimization. Int J Radiat Oncol Biol Phys. 1994; 30 (3): 699–706. 7928503 10.1016/0360-3016(92)90958-k

[7] Arnfield MR , Hartmann Siantar C , Siebers J , Garmon P , Cox L , Mohan R . The impact of electron transport on the accuracy of computed dose. Med Phys. 2000; 27: 1266–1274. 10902555 10.1118/1.599004

[8] de Jaeger K , Hoogeman MS , Engelsman M , et al. Incorporating an improved dose calculation algorithm in conformal radiotherapy of lung cancer: Re‐evaluation of dose in normal lung tissue. Radiother Oncol. 2003; 69: 1–10. 14597351 10.1016/s0167-8140(03)00195-6

[9] Budgell GJ , Perrin BA , Mott JHL , Fairfoul J , Mackay RI . Quantitative analysis of patient‐specific dosimetric IMRT verification. Phys Med. Biol. 2005; 50: 103–119. 10.1088/0031-9155/50/1/009 15715426

[10] Low DA , Harms WB , Mutic S , Purdy J . A technique for the quantitative evaluation of dose distributions. Med Phys. 1998; 25: 656–661. 9608475 10.1118/1.598248

[11] Depuydt T , van Esch A , Huyskens DP . A quantitative evaluation of IMRT dose distributions: Refinement and clinical assessment of the gamma evaluation. Radiother Oncol. 2002; 62: 309–19. 12175562 10.1016/s0167-8140(01)00497-2

[12] Suzuki H , Oshima H , Shiraki N , Ikeya C , Shibamoto Y . Comparison of two contrast materials with different iodine concentrations in enhancing the density of the aorta, portal vein and liver at multi‐detector row CT: A randomized study. Eur Radiol. 2004; 14 (11): 2099–2104. 15309493 10.1007/s00330-004-2439-5

[13] Hammer GM , Kuhn MJ , Meis DM , Meis LC . Quantitative contrast media dose evaluation for cranial computed tomography. Comput Med. Imaging Graph. 1995; 19 (3): 287–293. 10.1016/0895-6111(95)00006-c 7641173

[14] Wertz H , Jäkel O . Influence of iodine contrast agent on the range of ion beams for radiotherapy. Med Phys. 2004; 31 (4): 767–773. 15124994 10.1118/1.1650871

